# Pain Evaluation in the Paediatric Emergency Department: Differences in Ratings by Patients, Parents and Nurses

**DOI:** 10.3390/ijerph19042489

**Published:** 2022-02-21

**Authors:** Beata Rybojad, Daniel Sieniawski, Paweł Rybojad, Marzena Samardakiewicz, Anna Aftyka

**Affiliations:** 1 Clinic of Anaesthesiology and Paediatric Intensive Care, Medical University of Lublin, 20-093 Lublin, Poland; beata.rybojad@umlub.pl; 2 Department of Emergency Medicine, Medical University of Lublin, 20-081 Lublin, Poland; daniel.sieniawski@umlub.pl; 3 Chair and Department of Thoracic Surgery, Medical University of Lublin, 20-090 Lublin, Poland; pawel.rybojad@umlub.pl; 4 Chair and Department of Psychology, Medical University of Lublin, 20-081 Lublin, Poland; marzena.samardakiewicz@umlub.pl; 5 Department of Anaesthesiological and Intensive Care Nursing, Medical University of Lublin, 20-081 Lublin, Poland

**Keywords:** emergency department, pain scales, paediatric pain evaluation, self-report

## Abstract

The pain experienced by paediatric patients is rarely evaluated in emergency departments. The aim of the present study was to compare the degree of conformity in patients’ pain severity when assessed by themselves (if possible), their parents and a triage nurse trained in pain evaluation. Methods: A cross-sectional observational study was conducted at a tertiary paediatric emergency department in Eastern Poland involving children (aged six months to eighteen years), their parents and nurses. The patients had their pain assessed while collecting a medical history. For children ≥ four years of age, the Numerical Rate Scale was used by patients, parents and nurses to evaluate pain. Patients under four years of age were evaluated by parents and nurses using the FLACC scale. Results: Eighty patients and their parents were enrolled in the study. For children ≥ four years, patients rated their pain significantly higher than both their parents (*p* = 0.03) and nurses (*p* < 0.001), with the latter group producing the lowest scores. For children under four years of age, parental pain assessments did not significantly differ from those of nurses. Conclusion: Compared to the patients themselves and their parents, nurses tended to assign lower pain scores for children. Pain should be assessed on admission to the ED and, whenever possible, by the patients themselves.

## 1. Introduction

For many years, it was argued in the medical community that young children do not feel pain. This misconception was based on the idea that infants, especially neonates, have immature nervous systems, and that a complete myelination process was necessary for the functioning of neural pathways [1]. The objective assessment of pain severity in children is challenging for healthcare professionals. Paediatric patients with limited communication skills are unable to report their pain as “real” pain, and not just fear, discomfort or hunger. This explains why children are at risk of oligoanalgesia [2]. In many countries, paediatric pain in emergency cases is inadequately managed in spite of existing procedures [3,4].

Upon arrival, an accurate assessment of pain intensity, which is a subjective sensation, should be conducted. For pain evaluation, different scales are applied depending on the age of the patient, state of consciousness and availability. The intensity of pain can be estimated by individual self-report scales or, in younger children, by behavioural assessment. In emergency departments (ED), simple measurements should be used to elicit clear answers on the presence or the severity of pain. If a patient is conscious and communicative then pain can be measured using one of the popular pain scales, such as the Numerical Rating Scale (NRS), the Visual Analog Scale (VAS), the Face Pain Scale (FPS) or the Alder Hey Triage Pain Score (AHTPS) [5,6,7,8]. The utility of these tools varies based on the age of the child and the ability to respond to questions. Most children older than four years of age are able to estimate their pain intensity using these scales [9], and younger children may be assessed by the Face Legs Activity Cry Consolability (FLACC) scale [10]. 

Clearly, children suffering from pain should be provided with immediate pain relief. However, there are still questions regarding how to properly assess pain in children, what the ideal analgesics are and the doses that should be administered. In addition, there are typically problems with intravenous access in children, delayed transport to the hospital, fear of complications (especially after opioids) and patient refusal [11]. 

The objective of this study was to compare pain evaluations made by a child, a nurse and a parent. We hypothesised that pain scores measured by the parent and nurse should be similar as they are both “external bodies”. Moreover, we also wanted to examine the parental ability to assess a child’s pain using simple tools, and to compare these results with nurses’ and patients’ scores.

To our knowledge, this is the first study in Poland to compare pain evaluation in the ED between a child, parent and triage medical staff. 

## 2. Materials and Methods

### 2.1. Design

This cross-sectional observational study was conducted in a tertiary referral paediatric ED in Eastern Poland between March and July. The study involved children (aged 6 months to 18 years), their parents and triage nurses. Data were collected over eight night and day shifts (four during the week and four during the weekend).

### 2.2. Participants

The inclusion criteria included an age of under 18 years for the child, an ability of the legal guardian to speak, write and read in Polish and the willingness of the guardian to sign an informed consent form. Children ≥ 4 years old were included in the study if they were able to assess their pain using the NRS. The exclusion criteria included clinical instability or an illness necessitating urgent admission to the intensive care unit, developmental delays or neurologic impairments, any intoxication causing disturbances of consciousness (poisoning, alcohol, narcotic overdose, stimulants, etc.), an altered mental status, underlying chronic pain conditions (e.g., diabetes) or a medical history of multiple painful experiences (e.g., malignancies).

After the triage, while the children and their parents waited for contact with a physician, the researcher offered them participation in the study. After an informed written consent form was signed by the legal guardian (and a child between 16 and 18 years of age), a short questionnaire with questions on demographics, medications (including analgesics) and the reason for the ED visit was administered. Parents and their children were also trained on how to use the pain scales. In the next step, the researcher documented the results of pain assessments made by the parents and children ≥ 4 years of age. Data on the child’s pain assessment made by the nurse during the triage were also collected. Every nurse was trained in pain evaluation and had at least three years of work experience in the ED.

The STROBE checklist was utilised to ensure quality reporting during this study.

### 2.3. Instruments

The main outcome measures for this study were the pain scores produced by the nurses, parents and patients. Two pain scales were used depending on the patient’s age, either the FLACC or the NRS, both of which had total scores ranging from 0 to 10. These two scales were already being used in the hospital to evaluate postoperative pain in children, thus all nurses taking part in the study were familiar with these instruments (every nurse had to be trained on how to use these tests, no matter the department). The FLACC scale was used by the parents and the nurses to evaluate pain levels in children up to 4 years of age (Table 1), and the NRS was used by patients, parents and nurses for children aged 4 years and older. The following cut-off points were used for both scales: 1–3 points = mild pain (analgesia not compulsory), 4–6 points = moderate pain (analgesia should be administered) and 7–10 points = severe pain (opioid and/or local analgesia should be provided). For the FLACC scale, the points (0–2) for 5 observed activities are summed for the total score.

This behavioural tool was developed by Merkel and colleagues for children who are not able to verbalise the presence or severity of pain [10,12]. It is often used to assess pain in children between the ages of 2 months and 7 years, or in individuals that are unable to communicate their pain. The following instructions for the use of this scale (used with permission of The Regents of the University of Michigan) were applied in our research:

Patients who are awake: Observe for at least 1–2 min. Observe the legs and body uncovered. Reposition the patient or observe activity, assess the body for tenseness and tone. Initiate consoling interventions if needed.

Face: Score 0 points if the patient has a relaxed face, eye contact and interest in surroundings. Score 1 point if the patient has a worried look on their face, with eyebrows lowered, eyes partially closed, cheeks raised, or mouth pursed. Score 2 points if the patient has deep furrows in the forehead, with closed eyes, open mouth and deep lines around the nose/lips.

Legs: Score 0 points if the patient has usual tone and motion of the limbs (legs and arms). Score 1 point if patient has increased tone, rigidity, tenseness, or intermittent flexion/extension of the limbs. Score 2 points if the patient has hypertonicity, legs pulled tight, exaggerated flexion/extension of limbs, or tremors. 

Activity: Score 0 points if the patient moves easily and freely and has normal activity/restrictions. Score 1 point if the patient shifts positions, is hesitant to move, is guarding, has a tense torso, or pressure on a body part. Score 2 points if the patient is in a fixed position, rocking, has side-to-side head movement, or is rubbing body parts. 

Cry: Score 0 points if the patient has no cry/moan when awake or asleep. Score 1 point if the patient has occasional moans, cries, whimpers, or sighs. Score 2 points if the patient has frequent/continuous moans, cries, or grunts. 

Consolability: Score 0 points if the patient is calm and does not require consoling. Score 1 point if the patient responds to comfort by touch or talk in 0.5–1 min. Score 2 points if the patient does not respond to comfort by touch or talk.

Whenever feasible, the behavioural measurement of pain should be used in conjunction with self-report. When self-reporting is not possible, the interpretation of pain behaviours and decision-making regarding the treatment of pain requires careful consideration of the context in which the pain behaviours are observed. Each category is scored on the 0–2 scale, which results in a total score of 0–10. Assessment of the behavioural score: 0 = relaxed and comfortable, 1–3 = mild discomfort/pain, 4–6 = moderate discomfort/pain and 7–10 = severe discomfort/pain.

### 2.4. Statistical Analysis

Mean values (M) and standard deviations (SD) were calculated for the quantitative variables. The Wilcoxon signed-rank test (W) was used to assess differences between the pain intensity measurements made by the nurses, children and parents, and the Spearman rank coefficient was used to analyse the relationships between quantitative variables. A *p*-value ≤ 0.05 was considered statistically significant.

## 3. Results

### 3.1. Children > 4 Years Old

A total of 80 children were enrolled in the study. Of these participants, 50 were over 4 years old and were able to rate their own pain. The mean age of the patients was 12 years. Children with an injury predominated the sample (*n* = 36, 72.0%). The median (Q2) pain intensity in the nurse’s assessment was 5 points, which means that 50% of the children felt pain equal to or greater than 5 points on the NRS. According to the nurses, the 25th percentile (Q1) was 4 points and the 75th percentile (Q3) was 7 points. This means that 25% of the children experienced pain of no more than 4 points on the NRS, and a further 25% of patients, in the opinion of the nurses, felt pain at a level of 7 points or greater. Children assessed their pain level higher compared to parents and nurses: the 25th percentile (Q1) was 5.75 points, the median (Q2) was 7.5 points and the 75th percentile (Q3) was 9 points. Parents assessed their child’s pain level lower compared to the children’s assessment: the 25th percentile (Q1) was 5.0 points, the median (Q2) was 7.0 points and the 75th percentile (Q3) was 8.25 points (Table 2).

The mean pain intensity reported by the children (7.12 ± 2.12 points) was significantly higher than that reported by the nurses (5.26 ± 1.92 points; W = −5.221, *p* < 0.001). The mean pain intensity reported by the parents (6.56 ± 2.28 points) was significantly higher than that reported by the nurses (5.26 ± 1.92 points) as well (W = −4.484, *p* < 0.001). A smaller difference was observed in the intensity of the pain reported by the children (7.12 ± 2.12 points) compared to that reported by the parent (6.56 ± 2.28 points), although it was statistically significant (W = −2.185, *p* = 0.03; Table 3).

Pain intensity in the children’s assessments positively correlated with the severity of pain in the parent’s assessments (rho = 0.66, *p* < 0.01), and to a lesser extent with the severity of pain in the nurse’s opinion (rho = 0.53, *p* < 0.01). The correlation analysis also indicated that the age of the patient correlated positively with the severity of pain in the parent’s evaluations (rho = 0.30, *p* < 0.05), but not the patient’s own evaluations (Table 4).

### 3.2. Infants and Toddlers

Among children from birth up to the age of 4 years, non-traumatic illnesses predominated (*n* = 23, 76.7%) the sample and injuries were much less frequent compared to the older patients (*n* = 7, 23.3%). The median pain intensity in the nurse’s assessment was 5 points, the 25th percentile was 3.75 points and the 75th percentile was 7 points. Parents assessed their child’s pain level as slightly higher compared to the nurse’s assessment. The 25th percentile was 3.75 points, the median was 5.50 points and the 75th percentile was 7.25 points (Table 5).

The mean pain reported by the parents (5.37 ± 2.54 points) was not significantly different from that reported by the nurses (4.90 ± 2.67 points; W = −1.946, *p* < 0.052; Table 6).

The pain severity assessed by the parents of children under four years of age strongly correlated positively with the pain severity assessed by the nurses (rho = 0.78, *p* < 0.01; Table 7).

## 4. Discussion

In Poland, hospital departments that effectively monitor and treat pain are awarded a “Hospital without Pain” certificate every three years by the Polish Association for the Study of Pain [13]. Unfortunately, there are still “white places” where pain, which is important from a pathophysiologic point of view, is ignored and even considered necessary for a diagnosis. 

Overall, it was found that the pain assessment scores differed depending upon the scoring group. In particular, our findings show that nurses’ pain scores were significantly lower than those of children aged > 4 years and their parents. In addition, for older children, the nurses’ pain scores were significantly lower compared to the parental scores. For children aged < 4 years, pain scores between the nurses and parents did not significantly differ.

Only a few studies have directly compared pain assessment scores between children, their guardians and medical professionals [14,15,16,17,18]. However, the current results are in agreement with previous studies indicating that the pain intensity experienced by children is higher than that reported by parents and nurses [15,16]. For example, Batalha and Soussa examined pain scored by children, parents and nurses and reported that, regardless of the scales used (NRS and the FLACC scale), the scores from parents and nurses were always lower than those reported by children [19]. Another study compared pain scores between parents, physicians and children using different scales. Here, parents and physicians used the NRS, while children 3–8 years old used the FSP-Revised and those aged 8–15 years used the VAS. It was revealed that doctors underestimated pain compared to both the patients and the parents [16]. An additional study examining nurses’ evaluations of children’s pain also indicated that nurses scored pain lower than patients and their guardians; however, the differences in evaluation decreased when assessing fractures [19]. 

Zhou et al. also performed a systematic review and meta-analysis of twelve studies examining the relationship between self-report pain ratings for the dyads of child and parent, child and nurse and parent and nurse. The studies included in this review recruited children experiencing pain due to surgery or medical procedures. Of the twelve studies analysed, nine reported significant correlations between children and parents, eight between children and nurses and five between parents and nurses. The main conclusion of this survey was that parents’ and nurses’ perceptions of children’s pain should only be considered as estimates rather than expressions of the pain experienced, and that these estimates are not the same as children’s self-reports [20]. In general, these findings are in agreement with the current results. However, it must be emphasised that the sample sizes were relatively small in this study (e.g., the meta-analysis included only 12 studies). 

The current results also show that, for older children, parental pain scores were more closely related to the child’s score than to the nurses’ scores. Other studies have reported similar results. For example, Maciocia et al. have previously reported that parents assess pain at a level similar to their children. This may result from greater experience with one’s own family member, including his/her typical sensory or emotional states, even though they are not always able to differentiate pain from these states [14]. Another study has also shown that there are no significant differences between parent and child pain scores, and that a nurse’s score is significantly lower than both groups [17]. Similarly, Hla et al. have reported that, in communicative children following out-patient elective surgery, pain scores reported by the patients and their parents do not significantly differ. However, this latter study did observe significant differences between nonverbal children, parent and nurse ratings [18]. Canadian researchers also conducted interviews with nurses, physicians and parents whose children were admitted to an ED in severe pain, and barriers to improving the assessment and treatment of pain were explored. Parents felt that the self-reported pain score given by their child reflected their child’s level of pain [21]. Although our study was based on other methods, these results are in agreement concerning children’s and their parents’ pain assessments and the concordance between them. 

While the reasons for the differences in pain evaluation across the participant groups are unknown, they may relate to several factors. First, children are more prone to painful experiences, but they become even more vulnerable when they find themselves unable to orally express their pain [17]. In addition, professionals in paediatric EDs are used to taking care of children who suffer, as these departments are usually crowded with sick children. Additionally, ED personnel try to perform their duties as fast as possible as many patients are waiting for help. These factors can sometimes result in a lack of empathy in ED nurses, which may contribute to the underestimation of pain by those not emotionally involved in the suffering of a child. On the other hand, nurses have specific skills in the area of pain evaluation, so their estimates should better correlate with children’s self-reports than parents [17]. Moreover, professionals also know that a young child that is experiencing fear might behave as if they are in more pain. In such cases, the guardians and their children themselves are likely to overestimate the actual pain. Further research will be necessary to identify the factors that relate to the differences in evaluation across these participant groups.

Pain evaluation should occur routinely at the triage desk along with the measurement of other vital signs [2,13]. However, Stevens et al. reported that in only 28% of paediatric pain cases was pain documented and did children receive adequate pain management following a painful procedure [22]. In Poland, there is no compulsory pain assessment required in the ED (the ED in our hospital did not have such a procedure until recently) and not all centres follow their own pain procedures. Therefore, we are not able to compare the current results to those from other EDs as there is a lack of official data. However, a national survey conducted in Italian paediatric EDs showed that only 26% of departments used a routine pain assessment both at triage and in the ED [4]. 

The current study indicates that ED staff may need additional training with regard to pain evaluation in children [23] and the results suggest several avenues for improvement. In particular, the assessment of pain within a paediatric ED may be improved by better communication with the parents and children themselves, especially for older children. Zhou and Horgan claim that parents can play a significant role in pain evaluation, but it is possible that they may not realise minor ailments and the associated pain. Parents may apply a kind of “protection” whereby they only deal with pain they consider essential or significant [21]. In our study, parents were very surprised to be involved in their children’s pain assessments because they had never done this before. According to previous work, the parents’ role in helping medical staff to assess their children’s pain is significant [4], as it has been shown that healthcare providers sometimes do not know how to measure children’s pain, what tools should be used and the importance of analgesia [24]. 

In addition to improving communication, improved methods for assessing pain should be developed and implemented. To debunk the myths about pain in children (e.g., that “children tolerate pain better than adults, active children are not in pain, or that narcotics are dangerous medications for children and cause addiction”), some helpful tools have been developed. One of the comprehensive general approaches to pain assessment and management is QUESTT, which was developed by Baker and Wong and addresses many of the problems associated with pain evaluation in children [24]. For this method, the letter “Q” means “question the child” and the “U” stands for the use of pain rating scales. Changes in behaviour and physiological responses should also be evaluated (“E“), which is particularly important in preverbal or mentally delayed children. “S” stands for “sensitise parents”, stressing the critical role of parents in pain evaluation. As outlined above, even though medical staff may be more experienced, parents know their children best and can differentiate pain from fear or discomfort. Finally, action (e.g., administering analgesic medication) should be taken (“T”) followed by an evaluation of its results (“T”) [14,19]. Implementation of the QUESTT methodology may help to significantly improve pain assessments in the paediatric ED.

### Strengths and Limitations

One of strengths of our research is the fact that, in the group of patients older than four years of age, evaluation scores were directly comparable between parents, patients and triage nurses. We used the same pain scale across groups because it has been shown that, when all participants use the same assessment tool, the correlations are higher than when different scales are used [21]. However, our study has several limitations that must be considered. The main limitation lies in the small sample size; however, the sample was large enough for the statistical analyses performed. In Poland, it is difficult to convince parents to participate in studies, which is likely culturally conditioned. Another problem may be heterogeneity in the reasons for pain in our patients (e.g., traumatic vs. non-traumatic pain). Patients were also included in the study if they seemed that they were in pain at the time of the evaluation. However, it is possible that some patients were expressing their anxiety and not their pain.

## 5. Conclusions

The results of our study show that there are significant differences in pain evaluations among children, their parents and medical staff. Even parents underestimated their children’s pain, but their evaluation scores were closer to the child’s assessments. As such, nurses must avoid relying solely on parents’ interpretations or their own perceptions when assessing children’s pain, which can have important implications for analgesia. Good cooperation between medical staff, parents and paediatric patients is necessary for better pain evaluations, and it must be realised that children’s self-reports adequate to age and parents’ assessments are important for pain management in EDs. Improving the communication between healthcare providers, parents and children, and implementing integrative methods (e.g., QUESTT) to assess pain, are likely to help with pain evaluation and management in the paediatric ED. Further research on a larger group will be needed to more accurately pinpoint the factors contributing to the differences in pain assessment.

## Figures and Tables

**Table 1:** Description of the scores for each category of the Face Legs Activity Cry Consolability (FLACC) scale for the behavioural assessment of pain *.

Category	Scoring		
	0	1	2
Face	No particular expression or smile	Occasional grimace or frown, withdrawn, disinterested	Frequent to constant frown, clenched jaw, quivering chin
Legs	Normal position or relaxed	Uneasy, restless, tense	Kicking, or legs drawn up
Activity	Lying quietly, normal position, moves easily	Squirming, shifting back and forth, tense	Arched, rigid, or jerking
Cry	No cry (awake or asleep)	Moans or whimpers, occasional complaint	Crying steadily, screams or sobs, frequent complaints
Consolability	Content, relaxed	Reassured by occasional touching, hugging, or being talked to, distractible	Difficult to console or comfort
Each of the 5 categories are scored from 0 to 2, which results in a total score between 0 and 10.			

* Used with permission.

**Table 2:** Numerical Rating Scale pain scores as assessed by nurses, children and parents. Patients were ≥4 years of age.

	*n*	Min.	Max.	Q_1_	Q_2_	Q_3_	M	SD
Age (y)	50	4	17	9.00	12.00	16.00	12.22	3.77
Nurse’s assessment	50	1	9	4.00	5.00	7.00	5.26	1.92
Child’s assessment	50	1	10	5.75	7.50	9.00	7.12	2.12
Parent’s assessment	50	0	10	5.00	7.00	8.25	6.56	2.28

Q_1_—the 25th percentile, Q_2_—the 50th percentile, Q_3_—the 75th percentile, M—mean, SD—standard deviation.

**Table 3:** Differences in the intensity of pain assessed by nurses, parents and children. Patients were ≥4 years of age.

	Pain Intensity (M ± SD)	Wilcoxon Test Value	Significance
Child’s assessment	7.12 ± 2.12	W = −5.221	*p* < 0.001
Nurse’s assessment	5.26 ± 1.92		
Parent’s assessment	6.56 ± 2.28	W = −4.484	*p* < 0.001
Nurse’s assessment	5.26 ± 1.92		
Parent’s assessment	6.56 ± 2.28	W = −2.185	*p* = 0.03
Child’s assessment	7.12 ± 2.12		

M—mean, SD—standard deviation.

**Table 4:** Correlations between pain assessments for the various groups. Patients were ≥4 years of age.

	Age (y)	Nurse’s Assessment	Patient’s Assessment	Parent’s Assessment
Age (y)	x			
Nurse’s assessment	0.26	x		
Patient’s assessment	0.18	0.53 **	x	
Parent’s assessment	0.30 *	0.61 **	0.66 **	x

* *p* < 0.05, ** *p* < 0.01.

**Table 5:** FLACC pain scores as assessed by nurses and parents. Patients were <4 years of age.

	*n*	Min.	Max.	Q_1_	Q_2_	Q_3_	M	SD
Age (y)	30	0.5	4	1.00	2.00	3.00	2.03	0.96
Nurse’s assessment	30	0	10	3.75	5.00	7.00	4.90	2.54
Parent’s assessment	30	1	10	3.75	5.50	7.25	5.37	2.67

Q_1_—the 25th percentile, Q_2_—the 50th percentile, Q_3_—the 75th percentile, M—mean, SD—standard deviation.

**Table 6:** Difference in the FLACC pain scores reported by the nurses and the parents. Patients were <4 years of age.

	Pain intensity (M ± SD)	Wilcoxon Test Value	Significance
Parent’s assessment	5.37 ± 2.54	W = −1.946	*p* = 0.052
Nurse’s assessment	4.90 ± 2.67		

**Table 7:** Correlations between pain assessments by parents and nurses. Patients were <4 years of age.

	Age (y)	Nurse’s Assessment	Parent’s Assessment
Age (y)	x		
Nurse’s assessment	0.01	x	
Parent’s assessment	0.02	0.78 **	x

** *p* < 0.01.

## Data Availability

Data supporting reported results can be found in the Clinic of Anaesthesiology and Paediatric Intensive Care, Medical University of Lublin, Gebali str. 6, 20-093 Lublin, Poland.
